# EFFECTS OF THE WALK ON! PROGRAM ON PHYSICAL FUNCTION IN COMMUNITY-DWELLING OLDER ADULTS

**DOI:** 10.1093/geroni/igad104.1695

**Published:** 2023-12-21

**Authors:** Judy Foxworth, Allison Heinrich, Kynleigh Holley, Caleb Bauguess, Amanda Deputy, Lindsay Ludwig, Jo Ann Coco-Ripp, Barb Nicklas

**Affiliations:** Winston-Salem State University, Winston-Salem, North Carolina, United States; WFSM, Winston Salem, North Carolina, United States; WSSU, Winston Salem, North Carolina, United States; WSSU, Winston Salem, North Carolina, United States; WSSU, Winston Salem, North Carolina, United States; WSSU, Winston Salem, North Carolina, United States; Winston-Salem State University, Winston-Salem, North Carolina, United States; Wake Forest School of Medicine, Winston Salem, North Carolina, United States

## Abstract

The objective of this study was to determine the effects of the Walk On! program on pain, strength, gait speed, gait endurance, and balance in community-dwelling, African American older women. Data presented are from those who could ambulate greater than 200 feet independently and who completed one 11-week session of the Walk On! program offered at a local church. Participants walked laps around a large multi-purpose room and completed balance challenges and leg strengthening exercises. The participants completed a battery of physical functional tests including gait speed (self-selected and fast speed), 6-minute walk distance (6MWT), 5 sit to stand time (STS), timed up and go (TUG), and single leg stance (SLS). Participants who completed both the pre- and post-tests and at least 75% of the intervention sessions were included in the analysis (n=14). Paired sample t-test was used to determine significant differences in the physical function tests with alpha level set at 0.05. The mean age of the those who completed the study was 75.8 ± 7.3 years. Both the TUG (p = .018) and pain scores (p = .009) significantly improved. There was no significant improvement in other physical function measures. Older African American women who participated in a bi-weekly supervised walking program had improvements in functional mobility and reduced pain. Further research is necessary to generalize these results for all older adults.

